# Factors Influencing Goal Attainment in Patients with Post-Stroke Upper Limb Spasticity Following Treatment with Botulinum Toxin A in Real-Life Clinical Practice: Sub-Analyses from the Upper Limb International Spasticity (ULIS)-II Study

**DOI:** 10.3390/toxins7041192

**Published:** 2015-04-08

**Authors:** Klemens Fheodoroff, Stephen Ashford, Jorge Jacinto, Pascal Maisonobe, Jovita Balcaitiene, Lynne Turner-Stokes

**Affiliations:** 1 Department of Neurorehabilitation, Gailtal-Klinik, Hermagor 9620, Austria; 2 Faculty of Life Sciences and Medicine Faculty of Life Sciences and Medicine, Department of Palliative Care, Policy and Rehabilitation, King’s College London, London SE5 9PJ, UK; E-Mails: Stephen.ashford@kcl.ac.uk (S.A.); Lynne.turner-stokes@dial.pipex.com (L.T.-S.); 3 Regional Rehabilitation Unit, Northwick Park Hospital, London HA1 3UJ, UK; 4 Adult Rehabilitation Department 3, Alcoitão Medical Rehabilitation Centre, Estoril 2649 506, Portugal; E-Mail: jor.jacinto@netcabo.pt; 5 Medical Affairs, Ipsen Pharma, Boulogne-Billancourt 92650, France; E-Mails: pascal.maisonobe@ipsen.com (P.M.); jovita.balcaitiene@ipsen.com (J.B.)

**Keywords:** stroke, upper limb spasticity, botulinum toxin-A, goal attainment scaling (GAS)

## Abstract

In this *post-hoc* analysis of the ULIS-II study, we investigated factors influencing person-centred goal setting and achievement following botulinum toxin-A (BoNT-A) treatment in 456 adults with post-stroke upper limb spasticity (ULS). Patients with primary goals categorised as passive function had greater motor impairment (*p* < 0.001), contractures (soft tissue shortening [STS]) (*p* = 0.006) and spasticity (*p* = 0.02) than those setting other goal types. Patients with goals categorised as active function had less motor impairment (0.0001), contracture (*p* < 0.0001), spasticity (*p* < 0.001) and shorter time since stroke (*p* = 0.001). Patients setting goals for pain were older (*p* = 0.01) with more contractures (*p* = 0.008). The proportion of patients achieving their primary goal was not impacted by timing of first-ever BoNT-A injection (medium-term (≤1 year) *vs.* longer-term (>1 year)) post-stroke (80.0% *vs.* 79.2%) or presence or absence of severe contractures (76.7% *vs.* 80.6%), although goal types differed. Earlier BoNT-A intervention was associated with greater achievement of active function goals. Severe contractures impacted negatively on goal achievement except in pain and passive function. Goal setting by patients with ULS is influenced by impairment severity, age and time since stroke. Our findings resonate with clinical experience and may assist patients and clinicians in selecting realistic, achievable goals for treatment.

## 1. Introduction

Upper limb spasticity (ULS) is a common condition following stroke, which can be both painful and disabling [1]. Botulinum toxin A (BoNT-A) is established as a well-tolerated and effective treatment for reducing post-stroke spasticity [1,2,3]. Many clinical trials of BoNT-A have demonstrated improvements in muscle tone, but change at the functional level has been harder to demonstrate [1,2,4,5,6]. This is partly due the wide diversity in patient presentation and goals for treatment, as well as the failure of commonly used standardised measures to capture that diversity.

Person-centred goal setting, in which goals for intervention are tailored to the individual patient, is an important feature of stroke and neurological rehabilitation [7,8]. Goal Attainment Scaling (GAS) is a method that captures the extent to which a patient’s individual goals are achieved, allowing the differential achievement of several personal goals to be expressed as a standardised score. GAS is increasingly being used in the context of spasticity management [9,10] and has been shown to be sensitive to changes following focal intervention that are not detected by more global measures [10].

However, GAS is not a measure of outcome *per se*, but a measure of the achievement of treatment intentions. Goal attainment depends on both the patient’s ability to change and the clinician’s ability to predict the outcome and to negotiate goals that are realistic and achievable [11]. Their clinical reasoning must take into account both the reversibility of the presenting problem and any confounds such as severity, duration and irreversible features (e.g., fixed contracture or soft tissue shortening (STS)). Clinical trials provide little information to guide this reasoning as most exclude patients with contracture formation [4,10,12]. In clinical practice, clinicians recognise that BoNT-A treatment can often be effective in the treatment of long-term spasticity, even in the presence of contractures. However, this is best achieved when patients and treatment goals are selected appropriately [9] and the appropriate concomitant therapies are available [13].

The Upper Limb International Spasticity (ULIS) programme was established to describe real-life clinical practice and patient-centred outcomes in the treatment of post-stroke ULS with BoNT-A. The second stage of the programme (ULIS-II, NCT01020500) was a large, international, observational cohort study, conducted across 84 centres in 22 countries [14,15]. The primary objective of the ULIS-II study was to assess responder rates, as defined by the achievement of the primary person-centred goal using GAS, and to establish a consistent and reproducible approach to the recording of goals and goal attainment as measured by GAS [14]. Findings from the study showed that the most common primary goals were improvements in passive function (caring for the affected limb), active function (using the affected limb), reduction of spasticity-related pain and prevention of contractures, with these goals achieved by 72%–86% of patients [15]. Importantly, the achievement of goals related to pain and passive function showed the highest rates of achievement (84%–86%), suggesting that these may be “best response” goal areas for intervention with BoNT-A.

In this *post-hoc* analysis of the ULIS-II study, we further interrogate the dataset to determine the patient- and/or therapy-related factors that may influence goal setting and achievement rates, including baseline patient characteristics, the interval between stroke and first-ever BoNT-A injection, presence or absence of contractures and the type and intensity of therapeutic input (TI).

## 2. Aims and Objectives

The overall aim of this study was to investigate the factors influencing goal achievement in subgroups of patients receiving BoNT-A treatment for post-stroke ULS in the ULIS-II study.

Individual objectives were to:

(i) Compare the baseline characteristics of patients in each of three groups, based on their primary goal for treatment: (a) passive function, (b) active function or (c) pain, with those of patients with different primary goals, and to describe the specific nature of the goals achieved.
(ii) Compare the baseline characteristics of patients and examine primary goal achievement in relation to timing of first-ever BoNT-A treatment after stroke, whether initiated in the medium-term (≤1 year) or in the longer-term (>1 year) post-stroke.
(iii) Examine the impact of severe contractures on the achievement of primary goals.
(iv) Compare the impact of TI (comprising physiotherapy and/or occupational therapy between baseline BoNT-A treatment and follow-up) by examining primary goal achievement in patients receiving high intensity TI (>10 sessions) and low intensity TI (<10 sessions).

## 3. Results

A total of 468 participants were enrolled in the ULIS-II study, from which 12 were excluded to form the efficacy population (*n* = 456). Of those excluded, 11 were due to non-attendance at follow-up visit and one was due to a change in clinical treatment plan requiring injection of the lower, rather than the upper, limb. Demographics, baseline characteristics and BoNT-A dose range across the total population have been published previously [14,15]. Table 1 shows the breakdown of baseline characteristics across the three main groups of patients, whose primary goals related to passive function, active function or pain.

### 3.1. Passive Function as a Primary Goal

Of the 456 patients included in the efficacy population, 132 (28.9%) had a primary goal related to passive function and 324 patients had a primary goal in other areas. Those with primary goals in passive function were slightly older (mean difference, 2.9 years; *p* < 0.05) and more likely to be female (*p* < 0.01). They also had more severe motor impairment (*p* < 0.0001), contractures (*p* < 0.01) and spasticity (*p* < 0.05) at baseline (see Table 1), and their mean time since onset of stroke was greater, but this did not reach statistical significance (*p* = 0.07).

**Table 1:** Baseline characteristics including motor impairment score and composite contracture score for patient groups whose primary goals were passive function, active function and pain.

Parameters	Patients with primary goals in passive function *vs*. all other patients				Patients with primary goals in active function *vs.* all other patients				Patients with primary goals in pain *vs*. all other patients			
	Passive function	All other	Between group difference (95% CI)	*p*-value	Active function	All other	Between group difference (95% CI)	*p*-value	Pain	All other	Between group difference (95% CI)	*p*-value
	(*n* = 132)	(*n* = 324)			(*n* = 104)	(*n* = 352)			(*n* = 61)	(*n* = 395)		
Gender, % male	46	63	−17.1 pp (−27.6, −6.5)	**0.0012**	63	57	5.4 pp (−5.9, 16.7)	0.38	59	58	0.8 pp (−13.4, 15.0)	0.98
Age, years	58.7	55.8	2.9 (0.1, 5.6)	**0.039**	55.2	57.1	−1.9 (−4.9, 1.1)	0.21	60.6	56.1	4.6 (0.9, 8.2)	**0.01**
Time since onset of stroke, months, median	40	36.5	6.2 * (−0.6, 13.4)	0.07	26.8	43.5	−11.2 * (−19.3, −4.3)	**0.001**	41.7	37.2	−1.8 * (−10.8, 8.6)	0.73
**Motor impairment score** ^‡^	5.1	4.1	1.0 (0.8, 1.2) ^†^	**<0.0001**	3.5	4.6	−1.1 (−1.4, −0.9)	**<0.0001**	4.5	4.3	0.2 (−0.2, 0.5)	0.31
Arm raise	2.2	1.8	0.5 (0.3, 0.6)	**<0.0001**	1.5	2	−0.5 (−0.7, −0.3)	**<0.0001**	2	1.9	0.2 (−0.0, 0.4)	0.1
Hand function	2.8	2.3	0.5 (0.4, 0.6) ^†^	**<0.0001**	2	2.6	−0.7 (−0.8, −0.5)	**<0.0001**	2.5	2.5	−0.0 (−0.2, 0.2)	1
**Composite contracture score**	5.5	4.4	1.1 (0.4, 1.8)	**0.006**	2.9	5.2	−2.3 (−3.0, −1.6) ^†^	**<0.0001**	5.9	4.5	1.4 (0.4, 2.3)	**0.008**
Shoulder and elbow (proximal)	2.5	1.9	0.6 (0.2, 0.9)	**0.003**	1.2	2.3	−1.1 (−1.4, −0.8) ^†^	**<0.0001**	2.8	2	0.8 (0.3, 1.2)	**0.003**
Hand and wrist (distal)	3	2.5	0.5 (0.1, 1.0)	**0.02**	1.7	2.9	−1.2 (−1.6, −0.8) ^†^	**<0.0001**	3.1	2.5	0.6 (0.0, 1.2)	**0.04**
Composite MAS score **	11.7	10.8	0.9 (0.2, 1.6)	**0.02**	10	11.3	−1.3 (−2.1, −0.5)	**<0.0007**	11	11	−0.09 (−1.0, 0.8)	0.93

Data are means unless otherwise stated. pp, percentage points; CI, confidence interval; MAS, Modified Ashworth Scale. Two sided *p*-values were computed using a Wald asymptotic test of equality (sex), Student’s *t*-test (age), and Wilcoxon’s rank sum test (other parameters). * For the difference in medians, the Hodges-Lehmann estimate of the location shift is presented together with the corresponding asymptotic (Moses) confidence interval. ^†^ Satterthwaite’s approximation for degrees of freedom was used, since equality of variance could not be assumed for this parameter. ^‡^ Motor impairment score: limitation of arm raising/reaching and of hand function rated as none, mild, moderate and severe. Composite contracture score: severity of contracture or soft tissue shortening (STS), rated on a scale of 1–12, which included both proximal and distal contractures. ** Modified Ashworth Scale (MAS) score (sum of MAS scores for shoulder, elbow, wrist, fingers and thumb—scores of 1+ being entered as 1.5.

**Figure 1:**
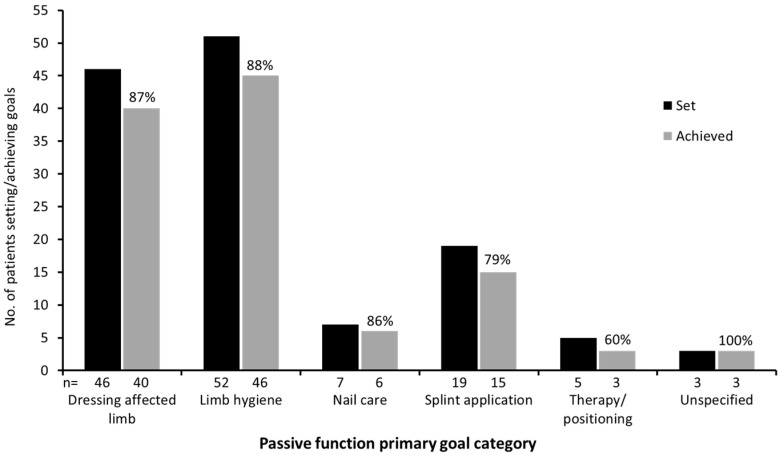
Task-based activities achieved for patients whose primary goals for treatment related to passive function. Unspecified goals related to “Improvement in ease of care” and “Less care burden”.

Rates of goal achievement were significantly greater for primary passive function goals (*n* = 113/132; 85.6%) than for other primary goals (*n* = 250/324; 77.2%; *p* = 0.04). Table S1 (Supplementary Information) provides a further breakdown of the types of passive function targeted. Among the specified goals, 63 (47.7%) were focused on the distal portion of the limb (*i.e.*, the hand/wrist), and nine (6.8%) were focused on the proximal portion (elbow/shoulder); the remaining 60 (45.5%) were focused on the whole arm or were otherwise unspecified, reflecting the wide range of muscles used in this cohort [15]. The achievement of five different types of task-based activities—dressing the affected limb; maintaining hygiene in the palm, axilla and elbow crease; nail cutting; splint application; therapy and/or positioning—is shown in Figure 1.

### 3.2. Active Function as a Primary Goal

There were 104 patients (22.8%) in the efficacy population who had a primary goal related to active function and 352 patients had a primary goal in other areas. There were no differences in age and gender between the two groups (Table 1). However, the patients with primary active function goals had significantly less severe motor impairment (*p* < 0.0001), contractures (*p* < 0.0001) and spasticity (*p* < 0.001) (see Table 1), and the time since stroke onset was shorter in this group (median difference 11.2 months (95% CI: 4.3, 19.3); *p* = 0.001).

**Figure 2:**
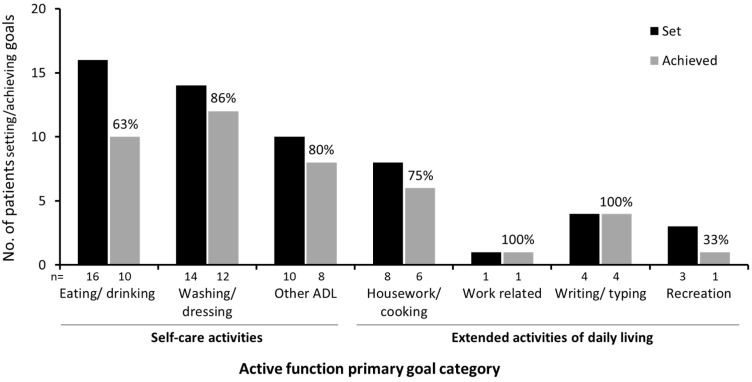
Task-based activities achieved for patients whose primary goals for treatment related to active function. (ADL, activities of daily living).

Rates of goal achievement were slightly lower for primary active function goals (*n* = 75/104; 72.1%) than for other primary goals (*n* = 288/352; 81.8%), but this difference did not reach statistical significance (*p* = 0.06). Table S2 (Supplementary Information) provides further breakdown of the types of active function goal achieved. In terms of the principal motor task involved, 83.3% of goals relating to grasp/hold tasks were achieved compared with only 57.1% of those relating to dexterity or reach. Figure 2 illustrates goal achievement in relation to the targeted functional activity. Over two-thirds of the goals (*n* = 40) were focused on self-care activities such as eating/drinking and washing/dressing, of which 30 (75.0%) were achieved, and the remainder of the goals (*n* = 16) were focused on extended activities of daily living, such as housework/cooking, work-related tasks, writing/typing and recreation.

### 3.3. Pain-Related Primary Goals

Sixty-one patients (13.4%) had a primary goal related to pain and 395 had primary goals in other areas. Baseline demographics were similar for the two groups except that patients with primary pain goals tended to be significantly older (*p* < 0.01) and had significantly more severe contractures (*p* < 0.01) at baseline (see Table 1). There was no difference in mean time since onset of stroke (*p* = 0.73).

Rates of goal achievement for primary pain goals (*n* = 51/61; 83.6%) were slightly higher than for other primary goals (*n* = 312/395; 79.0%), but again this difference did not reach statistical significance (*p* = 0.48).

### 3.4. Effect of Timing of First-Ever BoNT-A Treatment on Goal Attainment

Of the 456 patients in the efficacy population, 307 (67.3%) had received BoNT-A injection in the upper limb prior to ULIS-II (mean number of injection cycles, 6.4) and 149 (32.7%) were naïve to BoNT-A treatment. For this subanalysis, we examined how time elapsed between stroke and first-ever BoNT-A injection impacted on goal attainment in ULIS-II.

**Figure 3:**
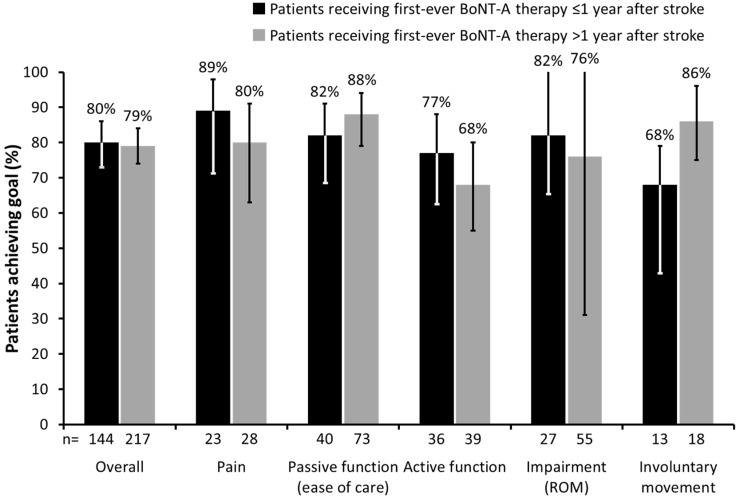
Proportion of patients who achieved their primary goal based on time from stroke to first-ever BoNT-A therapy. (Error bars correspond to lower and upper confidence limits of 95% CI. ROM, range of movement; CI, confidence interval).

Of the 454 patients with data available for this analysis, 180 (39.6%) received their first-ever injection ≤1 year after stroke, and 274 (60.4%) >1 year after stroke. The latter group was older (mean difference 2.8 years, *p* < 0.05), but the proportion of patients who achieved their primary goal was similar in the two groups (80.0% *vs.* 79.2%); *p* = 0.93 (Figure 3).

Although overall primary goal achievement was similar in both groups, patients receiving their first injection within the first year after stroke tended to achieve a greater proportion of goals related to active function (76.6% *vs.* 68.4%), pain (88.5% *vs.* 80.0%) and impairment (81.8% *vs.* 76.4%). In contrast, those receiving their first injection after one year were more likely to achieve goals in passive function (88.0% *vs.* 81.6%) and involuntary movement (85.7% *vs.* 68.4%).

### 3.5. Impact of Severe Contractures on Goal Attainment

**Figure 4:**
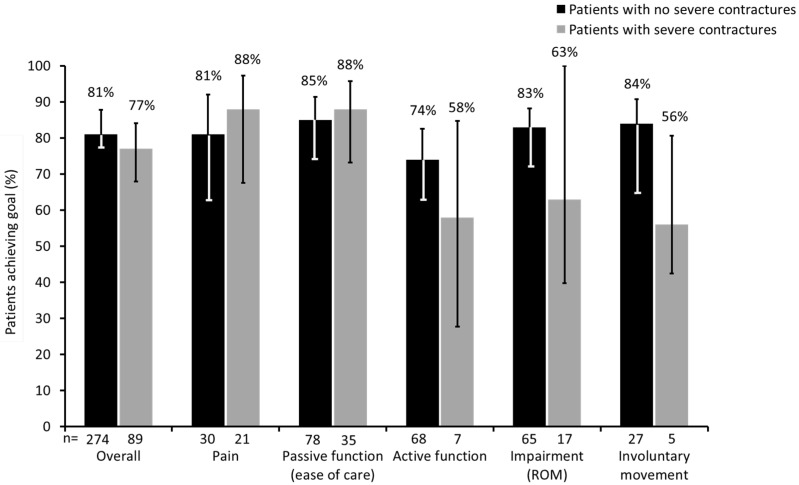
Proportion of patients who achieved their primary goal with BoNT-A therapy based on presence or absence of severe contractures. (Error bars correspond to lower and upper confidence limits of 95% CI. ROM, range of movement; CI, confidence interval).

Of the study population, 116 (25.5%) presented with severe contractures in at least one segment, compared with 340 (74.5%) patients with no or mild contractures. Patients with severe contractures were older than those with no contractures (mean difference: 3.3 years; *p* < 0.05) and also had a longer time since onset of stroke (difference in median number of months: 10.3 (95% CI: 1.8, 19.8); *p* ≤ 0.05). Overall, the two groups achieved a similar proportion of their primary goals (76.7% *vs.* 80.6%; *p* = 0.46). However, the goals were again somewhat different. Patients with severe contractures tended to achieve a greater proportion of goals related to pain and passive function whilst those without severe contractures were more likely to achieve goals relating to active function, improved passive range of motion and reduction of unwanted involuntary movements (Figure 4).

### 3.6. Impact of TI

As has been previously reported, the majority of patients in the ULIS-II study received some level of concomitant therapy, ranging from passive stretching (89.9%), exercise (78.9%), positioning (52.0%), splinting (32.2%), oral antispastic medication (28.5%), orthotics (20.2%) or functional electrical stimulation (12.7%) [15]. 

**Figure 5:**
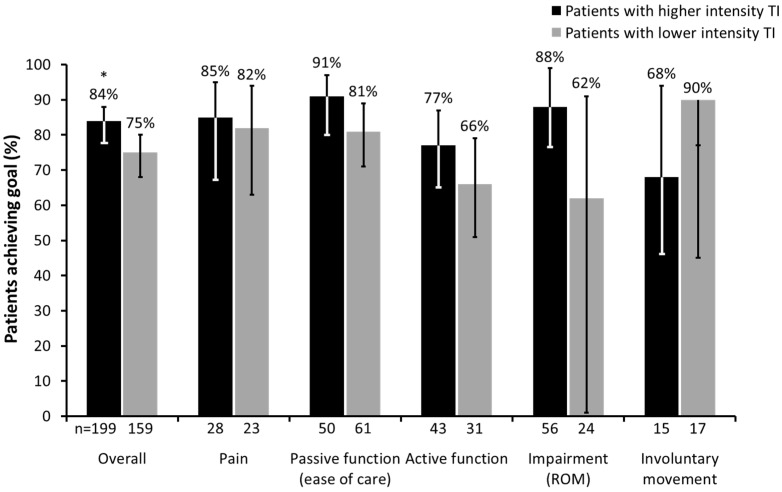
Proportion of patients who achieved their primary goal with BoNT-A therapy based on intensity of therapeutic input (TI). (* *p* < 0.05; Error bars correspond to lower and upper confidence limits of 95% CI. ROM, range of movement; CI, confidence interval).

Of the 451 patients evaluated in this sub-analysis (data unknown for *n* = 5), 238 (52.8%) patients received higher intensity TI (>10 sessions) and 213 (47.2%) received lower intensity TI (≤10 sessions) between BoNT-A treatment and goal evaluation at follow-up. Patients receiving higher intensity TI had a shorter time since onset of stroke (difference in median number of months: 11.4 (95% CI: 5.4, 18.1); *p* < 0.001). The overall rate of primary goal achievement was significantly greater in the higher intensity TI group (83.6% (199/238); 95% CI: 78.3%, 88.1%) than in the lower intensity TI group (74.6% (159/213); 95% CI: 68.3%, 80.3%; *p* < 0.05). The proportion of patients achieving their primary goals in both groups across the different goal categories are shown in Figure 5.

## 4. Discussion

ULIS-II was the first large, international, observational study providing data on the treatment of post-stroke ULS patients with BoNT-A in real-life clinical practice, in terms of goal setting, treatment planning and goal achievement. As reported in the primary ULIS-II study, the rate of patient goal achievement following BoNT-A therapy varied according to the type of primary goal, but was nevertheless high (>70%) across all categories investigated [15].

In this *post-hoc* analysis, we investigated the factors that influenced the selection and attainment of the primary goals for treatment in patients with ULS, using data from the ULIS-II study. Our findings suggest that primary goal setting was influenced by severity of motor impairment, contractures and spasticity, age and time since stroke, indicating that the right selection of the most appropriate goals is crucial to improving overall goal attainment and ultimately patient satisfaction with treatment. We also found that the overall success rate for primary goal achievement was not influenced by time elapsed from stroke to BoNT-A treatment or the presence of severe contractures, although trends emerged which suggest that these factors may influence certain types of goal achievement. The level of TI was found to influence goal achievement, with patients who received higher intensity therapy more likely to achieve their goal than those who received lower intensity therapy.

Moreover, further investigation of the specific task-based activities achieved by patients with an active primary goal suggests that BoNT-A treatment may increase independence for self-care and extended activities of daily living when used within a rehabilitation programme. In support of these findings, results from a randomised, placebo-controlled trial of BoNT-A in the treatment of post-stroke ULS demonstrated significantly greater levels of person-centred goal achievement with BoNT-A compared with placebo [10]. Furthermore, a recent meta-analysis of 10 clinical trials using BoNT-A indicates an overall improvement for adult ULS patients [16].

It has been demonstrated that intervention with BoNT-A early after stroke (2–12 weeks) can provide significant reductions in ULS [17] and also improve arm function [18]. Current guidelines also highlight the potential benefits of early treatment of ULS with BoNT-A [19]. However, current recommendations do not state an upper limit for timing of a point beyond which BoNT-A may be considered ineffective for overall reduction of baseline symptoms. In the ULIS-II cohort, only 40% of patients received their first-ever BoNT-A treatment within the first year after stroke. Reasons for a delayed start of ULS treatment in the other 60% remain unclear but younger age and more pain symptoms may be common factors triggering an earlier start of BoNT-A treatment in ULS. The timing of the first-ever BoNT-A injection following stroke did not impact the level of overall goal achievement in ULIS-II; however, it did appear to influence the types of goals achieved, with delayed start of first-ever BoNT-A treatment being more likely to result in goal achievement in the areas of passive function (ease of care) and involuntary movement. In contrast with the findings reported here, a recent prospective study performed in Austria and Germany found that time since onset of spasticity was a significant predictor of goal achievement (*p* = 0.03); however, in that study, goals were different from those in the ULIS-II study (as they included reduction in muscle tone and improvement in physiotherapy or occupational therapy, in addition to improvement of mobility, pain reduction and facilitation of care or hygiene of the affected limb). Additionally, outcomes were measured using a four-point scale of goal achievement, which may also have contributed to the different finding [20].

As noted in the introduction, patients with severe contractures are often excluded from BoNT-A trials, so an important new finding from this study was that patients with this level of contracture still achieved >85% of their goals where these were related to pain reduction and ease of care, although they achieved lower rates in other goal areas. These findings challenge the common perception that contractions are a contra-indication to BoNT-A indication. They demonstrate that patients with contractures may still benefit from BoNT-A treatment if the goals are appropriately selected. 

The majority of patients in the ULIS-II study received some level of concomitant therapy, ranging from passive stretching to functional electrical stimulation. This is consistent with current guidelines on treating ULS, which emphasise the combination of BoNT-A injections with concomitant therapies [3]. However, current recommendations are lacking on type and amount of concomitant therapies according to different levels of functioning and different goal areas. In this study, although we did not collect sufficiently detailed information to analyse how type of TI affected goal attainment, we were able to analyse the impact of intensity of TI on goal achievement with BoNT-A. Although our arbitrary cut-off of more or less than 10 sessions was a fairly simplistic way of analysing the effect of TI intensity on goal attainment, our finding that a significantly greater proportion of patients who received higher intensity TI achieved their goals compared with those who received lower intensity TI is an important one. Whilst the analysis does not provide evidence for cause and effect, the outcome adds further credence to the existing body of evidence that therapy inputs may be more critical for some goal areas than others. However, further research is required to elucidate the types and amount of therapy input that are likely to be most effective within the different goal areas.

This sub-analysis was based on a large, international, observational real-world study (ULIS-II) and had several limitations, including small numbers of patients in some goal areas and the relatively low frequency of reported impairments in areas such as cognitive and communication function, suggesting either selection bias, where these patients are less likely to be referred for treatment, or under-reporting. In addition, the data is based on only one injection cycle of BoNT-A [15].

However, the next stage of the ULIS programme (ULIS-III) will provide a larger, longitudinal dataset with several BoNT-A treatment cycles and detailed information about the types of concomitant therapy intervention, which should provide a more definitive description of the differential benefits of BoNT-A application when considering treating ULS patient subgroups with multiple and diverse treatment goals.

In conclusion, although the findings presented in this analysis will resonate with what is commonly observed in real-life clinical practice, they serve to provide an important body of exploratory data to confirm that experience. In this capacity, our findings will assist in designing future research which will help in selecting the right patients and appropriate goals for treatment with BoNT-A in ULS.

## 5. Materials and Methods

ULIS-II was an 18-month, post-marketing, international, multicentre, observational, prospective study, conducted at 84 centres across 22 countries in Europe, Pacific Asia and South America. The study was conducted in compliance with Guidelines for Good Pharmacoepidemiology Practices; ethical approval and written informed consent to the recording of anonymous data were obtained in countries where this was a requirement.

### 5.1. Study Population

Recruitment of patients took place between January 2010 and May 2011. The study included 456 post-stroke adults (≥18 years of age) with ULS, for whom a BoNT-A injection had already been planned and who had not received BoNT (A or B) within the preceding 12 weeks. Full details of the study, including rationale and methodology, have been published previously [14,15].

### 5.2. Measures

Principal measures used were:

(i) Achievement of the primary goals for treatment.
(ii) Motor impairment—limitation of arm raising/reaching and of hand function rated as none, mild, moderate and severe.
(iii) Composite contracture score—severity of contractures (STS), rated on a scale of 1–12, which included both proximal and distal contractures.
(iv) Modified Ashworth Scale (MAS) score (sum of MAS scores for shoulder, elbow, wrist, fingers and thumb—scores of 1+ being entered as 1.5).

The timing of the follow-up assessment to record goal achievement was at the discretion of the clinician, dependent on their normal practice and the type of goals set. The median follow-up time in this cohort was 14 weeks (range 2.6–32.3).

### 5.3. Post-Hoc Sub-Analyses

A *post-hoc* analysis of data from the ULIS-II study was performed, comprising three parts:

1. Analysis of patients’ baseline characteristics according to primary treatment goal, where this goal related to passive function, active function or pain. Passive function was described as the ability to care for the affected limb with assistance of a caregiver (e.g., washing, dressing, hygiene, nail care, positioning and splinting) while active function was defined as actively using the arm to perform specific tasks such as reaching, grasping, stabilising objects and fine hand use/dexterity. Descriptive summary statistics (*n*, mean, median) or frequency counts of demographic and baseline data (time since onset of stroke to visit 1 of ULIS-II, presence of fixed contractures and concomitant therapies) are presented for the efficacy population.
2. Analysis of GAS-defined achievement of primary treatment goals, where these related to passive function, active function or pain, as well as achievement of sub-goals within these areas.
3. Analysis of the impact of the following factors on goal setting and goal achievement:
  (i) Time elapsed since stroke to first-ever BoNT-A treatment, where medium-term was defined as first-ever BoNT-A treatment occurring ≤1 year after stroke and longer-term as first-ever BoNT-A treatment occurring >1 year after stroke.
  (ii) Presence of severe contractures (STS), defined as ≥three quarters limitation of movement in at least one segment (shoulder, elbow, wrist, hand).
  (iii) Intensity of TI, where high intensity TI was defined as more than 10 sessions of associated physiotherapy and/or occupational therapy between BoNT-A treatment and follow-up.

### 5.4. Statistical Analysis

Despite the large overall size of the study, the number of patients with primary goals within each area was relatively small. Therefore, the statistical analyses used were primarily descriptive and performed with the aim of identifying trends. For the analysis of baseline characteristics between different groups of patients, two-sided *p*-values were computed using a Wald asymptotic test of equality (sex), Student’s *t*-test (age), Wilcoxon’s rank sum test for other parameters with Satterthwaite’s approximation for degrees of freedom (when equality of variances could not be assumed for a parameter). Where median was calculated, the Hodges–Lehmann estimate of the location shift, with the corresponding asymptotic (Moses) confidence interval, was used. For the comparisons of primary goal achievement rates, a two-sided *p*-value was computed using a Wald asymptotic test of equality. For the relationship between achievement of pain-related primary goals and change in VAS rating of pain, MAS scores and patient and investigator-reported global benefit, two-sided *p*-values were computed using Cochran–Mantel–Haenszel tests.

## Supplementary Materials

Supplementary materials can be accessed at: http://www.mdpi.com/2072-6651/7/4/1192/s1.
