# Supplementary material for: Factors Influencing Goal Attainment in Patients with Post-Stroke Upper Limb Spasticity Following Treatment with Botulinum Toxin A in Real-Life Clinical Practice: Sub-Analyses from the Upper Limb International Spasticity (ULIS)-II Study
Source: Toxins (Basel). 2015 Apr 8;7(4):1192–205. doi: 10.3390/toxins7041192 (PMC4417963; doi:10.3390/toxins7041192)
Supplement: Supplementary file 1 [file toxins-07-01192-s001.pdf]

Supplementary Information

Table S1. Goal achievement by subcategories for patients with passive function as a primary goal.

Activities	Goal Set (n = 132)		Goal Achieved	
	N (%)	95% CI (%)	N (%)	95% CI (%)
**Injection area**				
Distal portion of the limb	63 (47.7)	39.0, 56.6	51 (81.0)	69.1, 89.8
Proximal portion of the limb	9 (6.8)	3.2, 12.5	8 (88.9)	51.8, 99.7
Whole arm or otherwise unspecified	60 (45.5)	36.8, 54.3	54 (90.0)	79.5, 96.2
**Subcategories**				
Dressing the affected limb	46 (34.8)	26.8, 43.6	40 (87.0)	73.7, 95.1
Limb Hygiene	52 (38.6)	30.3, 47.5	46 (88.2)	76.1, 95.6
Nail care	7 (5.3)	2.2, 10.6	6 (85.7)	42.1, 99.6
Splint application	19 (14.4)	8.9, 21.6	15 (78.9)	54.4, 93.9
Therapy/positioning	5 (3.8)	1.2, 8.6	3 (60.0)	14.7, 94.7
Unspecified	3 (2.3)	0.5, 6.5	3 (100.0)	29.2, 100.0

CI, confidence interval; Confidence intervals are computed using the Clopper–Pearson (exact) method. Percentages for goal set are based on the number of patients with a passive function primary goal. Percentages for goal achievement are based on the number of patients with a goal set in the area/subcategory.

Table S2. Goal achievement by subcategories for patients with active function as a primary goal.

Activities	Goal Set (n = 104) *		Goal Achieved	
	N (%)	95% CI (%)	N (%)	95% CI (%)
**Principal activity**				
Grasp/hold	18 (17.3)	10.6, 26.0	15 (83.3)	58.6, 96.4
Dexterity	21 (20.2)	13.0, 29.2	12 (57.1)	34.0, 78.2
Reach	7 (6.7)	2.7, 13.4	4 (57.1)	18.4, 90.1
Stabilising an object	1 (1.0)	0.0, 5.2	1 (100.0)	2.5, 100.0
Self-care activities (all)	40 (38.5)	29.1, 48.5	30 (75.0)	58.8, 87.3
Eating/drinking	16 (40.0)	24.9, 56.7	10 (62.5)	35.4, 84.8
Washing/dressing	14 (35.0)	20.6, 51.7	12 (85.7)	57.2, 98.2
Other activities of daily living (ADL)	10 (25.0)	12.7, 41.2	8 (80.0)	44.4, 97.5
Extended activities of daily living (all)	16 (15.4)	9.1, 23.8	12 (75.0)	47.6, 92.7
Housework/cooking	8 (50.0)	24.7, 75.3	6 (75.0)	34.9, 96.8
Work related	1 (6.3)	0.2, 30.2	1 (100.0)	2.5, 100.0
Writing/typing	4 (25.0)	7.3, 52.4	4 (100.0)	39.8, 100.0
Recreation	3 (18.8)	4.0, 45.6	1 (33.3)	0.8, 90.6

* Results show data for 104 goals set/103 patients due to missing data for one patient. ADL, activities of daily living. CI, confidence interval. Confidence intervals are computed using the Clopper–Pearson (exact) method. Percentages for goal set are based on the number of patients with an active function primary goal. For the subcategories of self-care activities, percentages for goal set are instead based on the number of patients with self-care activities. For the subcategories of extended ADL, percentages for goal set are instead based on the number of patients with extended ADL activities. Percentages for goal achievement are based on the number of patients with a goal set in the activity/subcategory.
